# Supplementary figures and images for: Identification of Novel Genes Associated with Partial Resistance to Aphanomyces Root Rot in Field Pea by BSR-Seq Analysis
Source: Int J Mol Sci. 2022 Aug 28;23(17):9744. doi: 10.3390/ijms23179744 (PMC9456226; doi:10.3390/ijms23179744)

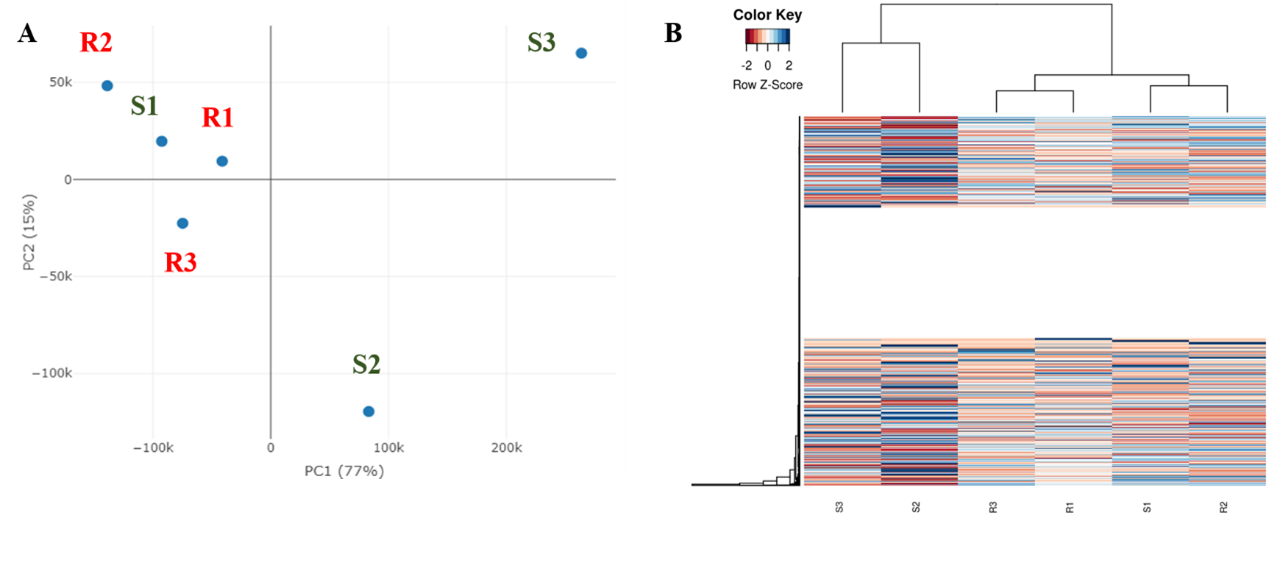

Supplement: Supplementary file 1 [file ijms-23-09744-s001.zip › Supplementary Fig. S1. PCA plot and hierarchical clustering plot of total examined genes by 3 biological replicates of R and S bulks.png]
